# The Role of Immunohistochemical Analysis as a Tool for the Diagnosis, Prognostic Evaluation and Treatment of Prostate Cancer: A Systematic Review of the Literature

**DOI:** 10.3389/fonc.2018.00377

**Published:** 2018-09-18

**Authors:** Arie Carneiro, Álan Roger Gomes Barbosa, Lucas Seiti Takemura, Paulo Priante Kayano, Natasha Kouvaleski Saviano Moran, Carolina Ko Chen, Marcelo Langer Wroclawski, Gustavo Caserta Lemos, Isabela Werneck da Cunha, Marcos Takeo Obara, Marcos Tobias-Machado, Adam G. Sowalsky, Bianca Bianco

**Affiliations:** ^1^Department of Urology, Hospital Israelita Albert Einstein, São Paulo, Brazil; ^2^Department of Pathology, Hospital Israelita Albert Einstein, São Paulo, Brazil; ^3^Department of Pathology, Rede D'OR São Luiz, São Paulo, Brazil; ^4^Department of Pathology, Hospital Israelita Albert Einstein, São Paulo, Brazil; ^5^Laboratory of Genitourinary Cancer Pathogenesis, Center for Cancer Research, National Cancer Institute, National Institutes of Health, Bethesda, MD, United States; ^6^Human Reproduction and Genetics Center, Faculdade de Medicina do ABC, Santo André, Brazil

**Keywords:** immunohistochemistry, prostate cancer, biomarkers, ERG, MYC, Ki-67, p53, PTEN

## Abstract

**Background:** Prostate cancer (PCa) is a heterogeneous disease that lends itself toward numerous therapeutic options depending on its risk stratification. One of the greatest challenges in PCa urologic practice is to select patients who should be referred for biopsy and, for those patients who are diagnosed with cancer, to differentiate between patients with indolent disease from those with an unfavorable prognosis and, to determine ideal patient management and avoid unnecessary interventions. Accordingly, there is a growing body of literature reporting immunohistochemical studies with the objective of determining a prostate cancer prognosis. Among the most frequent biomarkers studied are Ki-67, p53, PTEN, MYC, and ERG. Based on these findings, we systematically reviewed articles that assessed the role of these main prognostic markers in prostate cancer.

**Methods:** Consistent with PRISMA guidelines, we performed a systematic literature search throughout the Web of Science and PubMed Medline databases. We considered all types of studies evaluating the role of Ki-67, p53, PTEN, MYC, and ERG immunohistochemical analysis in prostate cancer until July 2017.

**Results:** We identified 361 articles, 44 of which were summarized in this review. Diagnostically, no single immunohistochemical marker was able to define a tumor as benign or malignant. Prognostically, Ki-67, p53, and MYC were related to the tumor grade given by Gleason score and to the tumor stage (higher levels related to higher tumor grade). Furthermore, Ki-67 was also related to higher PSA levels, shorter disease-free intervals and shorter tumor-specific survival; the latter was also related to p53. The loss of PTEN protein expression showed a higher association with biochemical recurrence and with a worse prognosis, beyond that predicted by the Gleason score and tumor stage. ERG staining also showed a strong association with biochemical recurrence.

**Conclusion:** There are several studies relating immunohistochemical markers with clinical-laboratorial outcomes in prostate cancer, the most frequent being Ki-67, p53, ERG, PTEN, and MYC. However, none of these markers have been validated by literary consensus to be routinely applied in medical practice.

## Introduction

Prostate cancer (PCa), excluding non-melanoma skin tumors, is the most prevalent cancer among men (1–3), with adenocarcinoma being the most frequent histological subtype. Despite the controversy (4), prostate-specific antigen (PSA) serum level (1, 3) screening routinely accompanies digital rectal examination. Diagnostic confirmation is accomplished by prostate biopsy guided by transrectal ultrasonography with or without magnetic resonance imaging (MRI) (5).

The heterogeneity of PCa histology was initially described by Donald Gleason in the 1960s and has improved over the years (6). Incorporating modifications to the Gleason grading system, the methodology used today is according to the International Society of Urological Pathology (ISUP) (7).

As a heterogeneous tumor which in turn allows a variety of therapeutic options, depending on its risk stratification, it is extremely important to identify the factors that determine PCa prognosis, thus defining the best course of clinical management. Significant prospective series show that radical treatment does not benefit low-risk patients according to the D'Amico's classification (8–10). Although the number of patients diagnosed with low-risk tumors and subjected to unnecessary surgery is substantial (11, 12).

Clinicopathological nomograms are commonly used to stratify risk, although with technological development and an increased understanding of tumor biology, immunohistochemical (IHC) and molecular biomarkers are emerging as powerful tools to distinguish tumors with distinct behaviors.

Immunohistochemical analysis consists of using monoclonal or polyclonal antibodies to detect specific antigens in tissue samples, and it is a widely used technique that can be applied in diverse situations, such as cellular differentiation, characterization of a tumor's primary site, detection of metastases, prognostic factors, as a predictor of targeted therapy response and even in the identification of structures, organisms and materials secreted by cells of interest. In prostate cancer, immunohistochemistry has an important role in the diagnostic confirmation of borderline cases due to the presence (or absence) of basal cells, detected by specific antibodies against it combined with racemase expression in luminal epithelial cells. Currently, the identification of biomarkers capable of predicting the course of the disease has been gaining importance (12–14).

Among the most frequent biomarkers studied that are associated with PCa are p53 (tumor protein p53), Ki-67 (marker of proliferation), ERG (ETS-related gene), MYC (proto-oncogene), and PTEN (phosphatase and tensin homolog), which are implicated in the control of cell proliferation and differentiation, angiogenesis, and apoptosis (15–25).

In the main series of active surveillance, approximately 30% of patients are reclassified and subjected to radical therapy. Immunohistochemical markers could be useful for the initial evaluation and for identifying cases with major or minor potential of progression, helping the decision process of whether to start a radical treatment. Another important application would be in patients with a low volume of ISUP 2, for which immunohistochemistry (IHQ) could be useful in selecting candidates for active surveillance.

Based on these findings, we systematically reviewed articles that studied the role of these main IHC prognostic markers in prostate cancer.

## Methods

The systematic review was performed in accordance with the Preferred Reporting Items for Systematic Reviews and Meta-Analysis (PRISMA) guidelines (26).

### Search strategy

A computerized literature search of PubMed to identify title and abstracts published was performed. The search was performed with and without MESH terms (Ki-67, p53, PTEN, MYC, ERG, immunohistochemistry, and prostate cancer). All references in the selected articles were checked, including hand-typed searches.

### Study eligibility

The final articles were selected based on the following set of inclusion criteria: (i) examined the association of immunohistochemical expression of Ki-67, p53, PTEN, MYC, and/or ERG with clinical outcome of prostate cancer; (ii) in humans; (iii) non-metastatic prostate cancer; (iv) articles published from 1966 until July 2017. No restriction was made regarding the study type or language. Articles were excluded for any of the following reasons: (i) could not be accessed in its entirety; (ii) were duplicated; (iii) were a review article.

Initially, titles were reviewed to assess whether they met the inclusion criteria. If, after assessing the abstract, there was any doubt regarding whether it met the relevant criteria, it was kept for more thorough, subsequent assessment. The list of potential articles was further shortened by performing detailed evaluations of the methods and results of each remaining paper.

We included patients with localized or locally advanced prostate cancer submitted for or in the process of definitive treatment. None of these patients were submitted to radiotherapy or hormonal therapy before biopsy or prostatectomy.

### Data collection

The following details were recorded for each study: author, year of publication, country where study was performed, study design, number of patients, population/setting, type of material used, main objective of the study (diagnostic and prognostic evaluation) and outcome reported (Gleason Score, PSA level, tumor stage, lymph node stage, tumor diameter, and Gleason upgrading).

## Results

**Figure 1:**
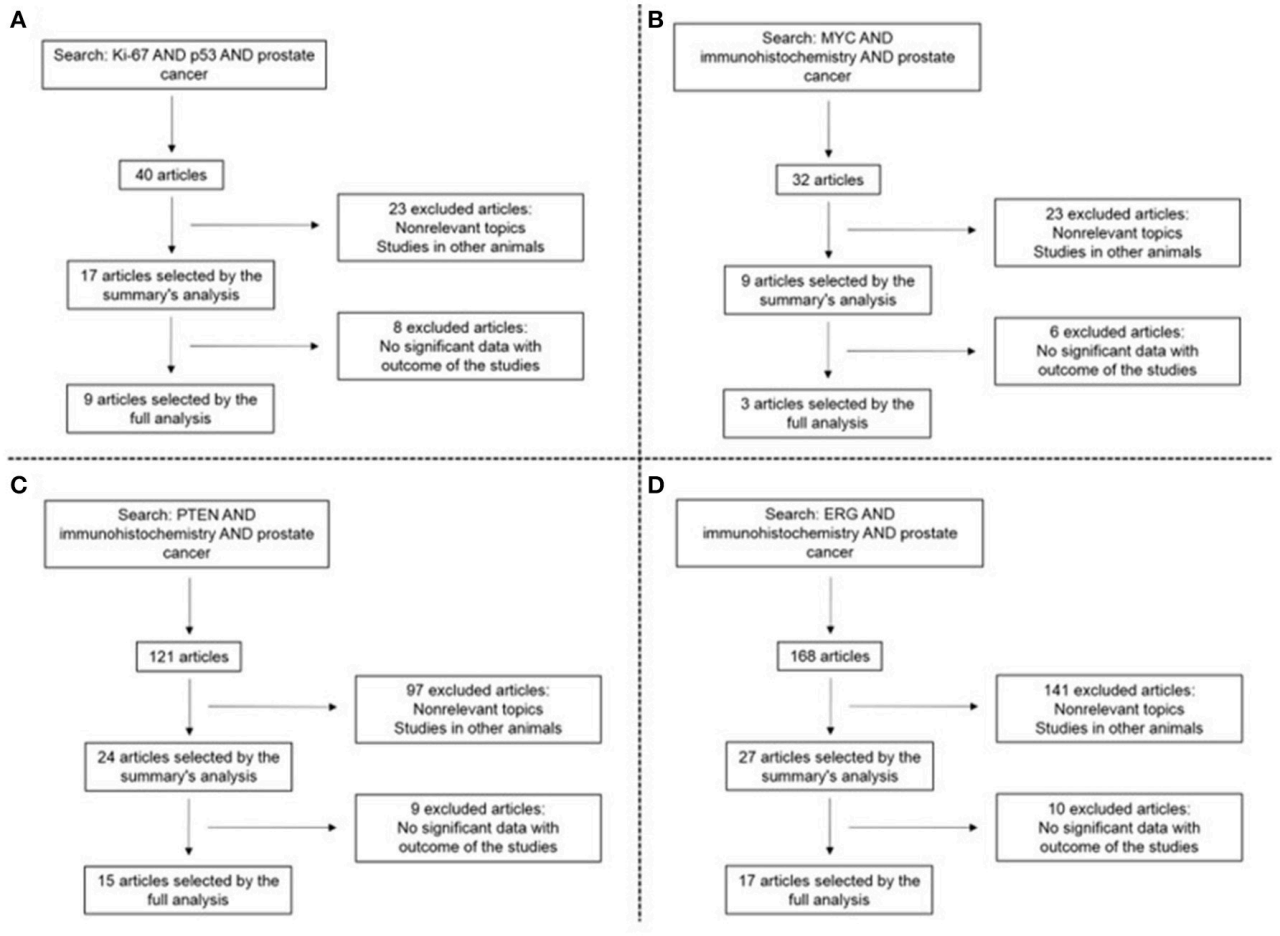
Search results concerning Ki-67 and p53 **(A)**, MYC **(B)**, PTEN **(C)**, and ERG **(D)**.

The literature search identified a total of 361 studies. Using the above inclusion and exclusion criteria a total of 42 studies were included in the systematic review: 9 concerning Ki-67 and p53; 3 concerning MYC; 15 concerning PTEN, and 17 concerning ERG. (2 studies concerned more than one marker). A schematic of the search is depicted in Figure 1.

The attachment archive depicts the characteristics of the studies included in systematic review based on the association of the immunohistochemical expression of Ki-67, p53, PTEN, MYC, and/or ERG with the clinical outcomes of prostate cancer (Supplementary Table 1).

Table 1 lists the main outcomes and studies, according to the studies that analyzed biopsy specimens and prostatectomy specimens.

**Table 1:** Immunohistochemical marker role in predicting outcomes in biopsy and surgical specimen analysis.

	**ERG**	**PTEN**	**MYC**	**Ki-67**	**p53**
BIOPSY SPECIMEN					
Gleason score	~	✔(27–29)	~	✔(30, 31)	✔(31, 32)
PSA level	✔(33)	~	~	✔(30)	✔(30)
T-stage	~	✔(27, 34, 35)	✔(36)	✔(32)	✔(32)
N-stage	~	~	~	✔(32)	~
Clinical insignificance	~	✔(28)	~	✔(30)	~
Tumor diameter	~	~	~	~	~
Gleason upgrading	~	✔(35,37)	~	✔(32)	~
Disease-free interval	~	✔(28, 35, 38, 39)	~	~	~
Overall survival	~	~	✔(36)	~	~
Tumor-specific survival	~	~	~	✔(31)	✔(31)
SURGICAL SPECIMEN					
Gleason score	✔(40–45)	✔(28, 46-49)	✔(50)	✔(51–53)	✔(32, 51, 52, 54)
PSA level	✔(33, 40, 44, 45, 55, 56)	✔(48)	~	~	~
T-stage	✔(25, 57-59)	✔(34, 46-49, 60)	✔(25, 36, 50)	✔(32, 51-53)	✔(32)
N-stage	~	✔(48)	~	✔(32, 51)	~
Clinical insignificance	~	✔(28)	~	~	~
Tumor diameter	~	~	~	~	~
Gleason upgrading	~	~	~	✔(32)	~
Disease-free interval	✔(40, 57, 61-63)	✔(28, 38, 46, 47, 49, 60, 64)	~	✔(32, 53, 65)	✔(65, 66)
Overall survival	✔(43)	✔(48)	✔(36)	✔(53)	✔(53)
Tumor-specific survival	✔(38)	✔(48)	~	✔(53)	✔(53)

*✔, Studies showed correlation; ~, No studies were found with this analysis*.

(n), Studies in accordance to the references

### Diagnosis

The vast majority of prostate cancer diagnoses are based on morphological assessment. IHC is complimentary in case of doubt, but its interpretation should always be evaluated in conjunction with histomorphology. None of the IHQ markers used with a diagnostic purpose (p63, high molecular weight keratins, racemase and ERG) are specific and/or sensitive enough to classify a case as benign or malignant.

### Prognosis

#### Ki-67

Ki-67 is a protein expressed in the nucleus of proliferating cells during mitosis interphase. It is expressed during late G1, S, G2, and M phases, but not during G0 phase (15). The Ki-67 protein is a cellular proliferation marker (16, 17) and it is widely used given its high reproducibility (15).

Of the selected articles, the analyzed materials in studies were from: prostatectomy specimens (5 studies) (32, 51–53, 65), biopsy specimens (3 studies) (30–32), and transurethral resections (TUR) (2 studies) (52, 53).

Chevrier et al. (51) quantitatively and positively correlated Ki-67 expression based on the H-score and tumor grade given by the Gleason score: H-score 2, 21, and 52 for Gleason score (3 + 3 = 6, 3 + 4 = 7, and 4 + 4 = 8), respectively (*p* = 0.0002). Other studies also showed significant statistical correlation between Ki-67 and the Gleason score (30, 31, 52, 53), of which 2 were biopsy specimens (30, 31) and the others were prostatectomy and/or TUR specimens (52, 53).

Ki-67 protein overexpression was also shown to be related to T stage and lymph node invasion (32, 51). Its higher expression was more related to stage T3 than to T2 (*p* = 0.0001), and found more in N1 (*p* = 0.0389) the latter also shown in Zellweger et al. (32). Zellweger et al. (32) showed that Ki-67 >10% (high) was associated with adverse pT stage (*p* = 0.036), specifically to seminal vesicle invasion (pT3b) with OR = 12 (2.5–57.0, 95% CI), *p* = 0.002.

Elevated Ki-67 showed an association with PSA serum levels for PSA ≤ 2.5 ng/ml compared with PSA between 10 and 20 ng/ml (*p* < 0.05) (30). In turn, low levels of Ki-67 were related to clinically insignificant tumors [determined by the Epstein criteria (67) and by PRIAS (68)], at *p* = 0.05 and 0.005, respectively (30).

In a study comparing biopsy and prostatectomy specimens, an elevated Ki-67 (>10%) in the biopsy was related to a higher Gleason score in the prostatectomy specimen (*p* = 0.001) (32). In the same study, an association between Ki-67 and the disease/biochemical recurrence-free interval was found, as also shown by Inoue et al. (65).

In general, an association between elevated Ki-67 and a higher percentage of tumor cell involvement on biopsy (*p* = 0.037), as well as shorter tumor-specific survival (*p* = 0.0007), can be found in the literature (31).

#### p53

Tumor protein 53 (p53) regulates the control of cell cycle progression and cell proliferation, as well as the intrinsic mitochondrial apoptosis pathway (16, 18). p53 acts as a transcription factor, controlling genes to prevent cell proliferation after DNA damage (19). When mutations in the *TP53* gene occur, cells that contain damaged DNA are not repaired nor are cells destroyed, resulting in the inability to stop the cell cycle or to initiate apoptosis, thus allowing the emergence of malignant cells (20). Mutations in *TP53* occur in more than 50% of malignancies, including high grade prostate cancers (21).

Of the selected articles, the analyzed materials in the studies were: specimens from prostatectomies (7 studies) (32, 51–54, 65, 66), biopsies (3 studies) (30–31), and TUR (2 studies) (52, 53).

Regarding p53 as an immunohistochemical marker, the relationship between its expression and tumor grade (Gleason score) was reported in various studies (32, 51, 52, 54). Chevrier et al. (51) showed this relationship in a quantitative manner using the H-score system, comparing histologic groups as follows: Gleason 3 + 3 had an H-score of 3, Gleason 3 + 4 had an H-score of 65 and Gleason 5 + 4 H-score = 195 (*p* = 0.0430) (51).

Zellweger et al. (32) demonstrated the relationship between p53 positivity (>20% of highly reactive nuclei) and seminal vesicle invasion (*p* = 0.001) (32). Nagao et al. showed a relationship between p53 and the PSA level (comparing PSA ≤ 2.5 ng/ml and PSA between 10 and 20 ng/ml) (*p* < 0.05) (30).

An association between p53 and biochemical recurrence free survival was shown in two studies, Inoue et al. (65) and Osman et al. (66) (*p* = 0.0097 and *p* < 0.01, respectively) (65, 66). In survival studies, p53 overexpression predicted shorter tumor-specific survival (*p* < 0.05) (53) and overall survival (*p* = 0.024) (31).

#### ERG

*ERG* (ETS-related gene) expresses an essential protein for the maintenance of vascular integrity. It is associated with cellular structure maintenance, and its loss leads to higher endothelial permeability. It is also associated with haematopoiesis, maintenance of platelets in peripheral blood and pre-cartilage formation (in mesoderm cells). Typically, it is not present in normal prostatic tissue. The protein is overexpressed in prostate cancer when the androgen-induced *TMPRSS2 (transmembrane serine protease 2)* gene fuses to the *ERG* gene (22).

Of the selected articles, the analyzed materials in the studies were specimens from prostatectomies (17 studies) (25, 33, 38, 40–45, 55–59, 61–63), TUR (3 studies) (33, 38, 59), biopsies (2 studies) (38, 33).

In the analysis of reviewed articles, there was a consistency between the relationship of ERG protein expression and the biochemical recurrence of PCa (38, 40, 57, 61–63). Hagen et al. showed that 13 of 28 patients (46.4%) who had surgical specimens with high ERG levels developed recurrence compared to only 3 (12%) of 25 patients who had tumors with low ERG levels (*p* = 0.006) (57). Font-Tello et al. also showed this association, with PSA progression observed in 3 of 25 (12%) ERG-negative patients compared to 13 of 38 (34.2%) ERG-positive patients (*p* = 0.04) (61). Kim et al. presented a relative risk for biochemical recurrence in ERG-positive patients of 8.964 (*p* = 0.002) (40).

An association between ERG-positive immunohistochemistry and Gleason score was frequently reported (41–45). Suh et al. (41) showed that ERG protein expression was more frequently detected in the subgroup with a lower primary Gleason grade (less than or equal to 7) than in the subgroup with a higher Gleason grade (*p* = 0.011) (41). Kron et al. (42) obtained similar results, with ERG positivity found more frequently in Gleason score 6 and 7 tumors, whereas a Gleason score of 8 to 10 displayed a lower positive frequency (*p* < 0.01) (42).

For PSA levels, ERG-positive cases were associated with lower preoperative PSA compared to ERG-negative cases (33, 40, 44, 45, 55, 56). Brooks et al. (45) showed a mean PSA of 7.9 ng/ml in patients with positive ERG expression and a mean PSA of 9.3 ng/ml in ERG-negative patients (*p* = 0.0003) (45). Kim et al. compared a group of patients with PSA serum levels lower than 10 ng/ml to a group of patients with PSA higher or equal to 10 ng/ml and showed that the relative risk for positive ERG protein expression was 4.3 favorable for the group with lower PSA levels (*p* = 0.039) (40).

For the presence of adverse pathologic features, Udager et al. (25) showed that ERG-positive tumors were associated with extraprostatic extension (*p* = 0.02). They also showed that these tumors were associated with elevated pathological stage greater than or equal to pT3 (*p* = 0.035) (25). This correlation was also reported by other groups (58, 59, 61).

Finally, several reports indicated that ERG-positive patients were enriched for PCa diagnosis at a younger age (44, 45, 55, 59). Schaefer et al. showed a mean age of 60 years for ERG-positive tumors in contrast to 63 years for ERG-negative tumors (*p* < 0.0001)(44). Similarly, Brooks et al. showed a mean age of 60.5 vs. 62.5; *p* < 0.0001, with the ERG-positive expression group having a younger age (45).

#### PTEN

*PTEN* (phosphatase and tensin homolog) is a tumor suppressor gene. It is a lipid phosphatase and a negative regulator of the PI3K/AKT/mTOR pathway, which controls cellular processes such as survival, proliferation, metabolim, migration and cellular architecture. The PTEN protein is frequently absent in some cases of prostate cancer, indicating a loss of function (23).

Of the selected articles, the analyzed materials in the studies were: specimens from prostatectomies (10 studies) (27, 28, 34, 38, 46–49, 60, 64), biopsies (6 studies) (28, 29, 35, 37–39), and TUR (3 studies) (34, 38, 69).

One of the most consistent correlations found in the literature was between the loss of PTEN and a higher recurrence rate or shorter disease-free interval, with the finding that PTEN is considered a biochemical recurrence predictor (28, 34, 38, 39, 46, 47, 60, 64). Murphy et al. showed that among Gleason score 7 or higher tumors, those with PTEN loss had a recurrence rate of 80% compared to 55% in those with intact PTEN (28).

In addition, a correlation between loss of PTEN staining and a worse disease prognosis was frequently observed. Studies revealed a higher risk of death among patients with prostate cancer and loss of PTEN staining compared to patients with proficient and/or partially reduced PTEN protein staining (48, 49, 69). Lahdensuo et al. presented a hazard ratio for risk of death by prostate cancer of 2.156 (95% CI 1.169–3.976, *p* = 0.014) in a univariate analysis that compared those with a total loss of PTEN to those with partial or no loss of PTEN (48).

Lotan et al. reported a relationship between the loss of PTEN protein and Gleason upgrading from biopsy to prostatectomy. They found that 18.3% (13/71) of tumors that had Gleason upgrading presented PTEN protein loss compared to 7% (7/103) of those without Gleason upgrading (*p* = 0.02) (37). Guedes et al. also showed this correlation (35).

PTEN loss was associated with extraprostatic extension in numerous studies (35, 46, 47), notably seminal vesicle invasion (46, 47, 60). It also showed an association with a higher Gleason score (27, 29, 46–49). Genomically, homozygous loss of *PTEN* was present in only 4% of patients with Gleason score 6 compared to 18% of those with a Gleason score of 8 to 10 (*p* < 0.0001) (47).

#### MYC

MYC is a nuclear transcription factor related to regulation of the cell cycle progression, metabolism, ribosome biogenesis, protein synthesis, mitochondrial functions and stem cell self-renewal. Its protein is frequently overexpressed in prostate cancer and can have a role in tumor initiation and/or progression (24, 25).

Of the selected articles, the analyzed materials in the studies were specimens from prostatectomy (3 studies) (25, 36, 50) and biopsies (2 studies) (25, 36).

In our literature review, we found correlations between MYC and the Gleason score. Prowatke et al. (50) showed that a decrease of MYC protein expression was related to an increase in Gleason score. MYC expression was 74, 54, and 28% in tumors with Gleason score 6, 7 and 8–9, respectively (*p* = 0.001) (50).

A correlation between a reduction of MYC protein expression and an increase of T-stage was also demonstrated, with 73% in pT2 and 36% in pT3–4, (*p* = 0.001) (50). These findings were also reported by Zeng et al. (36) (*p* < 0.001). Udager et al. demonstrated that MYC overexpression was related to the presence of extraprostatic extension (*p* = 0.004) in a multivariate analysis with HR = 5.780 (95% CI 2.125 to 15.722) (*p* < 0.001) (25).

## Discussion

Prostate cancer screening is constantly questioned because large studies failed to demonstrate any survival benefit (70–72) and because the overdiagnosis caused by the detection of indolent tumors leads to overtreatment and a worsened quality of life because of the treatment (73).

Although active surveillance should remain the preferred option in managing very low-risk and the majority of low-risk prostate cancers (74), it is constantly under-used in clinical practice worldwide. According to the US National Cancer Data Base, in 2013, less than 20% of men with low-risk prostate cancer were managed conservatively (75).

Active surveillance presents a cancer-specific survival similar to active treatment in very low and low-risk PCa patients. However, the disease progression-free survival rate is significantly higher in active surveillance patients, compared to men treated with radical prostatectomy or radiation therapy (9). Therefore, it is of foremost importance to determine whether there are any other factors that could improve the selection of patients that can be safely managed without definitive treatment.

For this, several IHC markers, are readouts for tumor behavior and were used to better define the prognosis and course of management for men with prostate cancer. However, there remains no literary consensus about which markers are more reliable for those purposes. In this manuscript, we emphasized what we believe to be the most promising markers, p53, Ki-67, MYC, ERG and PTEN, analyzed in both the biopsy and post-prostatectomy setting.

In the diagnostic scenario, in this systematic review, no immunohistochemical marker was found to be significant for diagnosing or defining a tumor as benign or malignant.

By contrast, for determining prognosis, immunohistochemical markers stand out in several studies, some presenting with consistent results. Ki-67, p53, and MYC were consistently related to the tumor grade given by Gleason score and to the tumor stage (higher levels related to higher tumor grade). Ki-67 was also related to higher PSA levels, shorter disease-free interval and shorter tumor-specific survival. Additionally, data showed a relationship between p53 and shorter tumor-specific survival. The loss of PTEN protein expression was related to biochemical recurrence and a worse prognosis, beyond Gleason score and tumor stage. One of the most consistent findings was the association between ERG staining and biochemical recurrence.

Considering all these findings in this systematic review, we propose that immunohistochemical markers are in the process of becoming consistent prognostics tools in clinical practice. As a scientific approach, the relationship between these markers and certain outcomes in prostate cancer is increasingly shown. However, gaining statistical significance and strength in clinical practice remains necessary.

Although cost-effective analyses are missing, a wider utilization of IHC markers in daily routine practice could facilitate decision-making by the clinician and even encourage the patient to follow the most appropriate path for disease management.

## Conclusion

In summary, there are several studies relating immunohistochemical markers with clinical-laboratory outcomes in prostate cancer, the most frequent being Ki-67, p53, ERG, PTEN, and MYC. However, none of these markers have been validated and, consequently, they cannot be applied in medical practice.

Positive staining for Ki-67, p53 and MYC were related to higher tumor grade and stage. Ki-67 was also related to PSA levels, disease-free interval and tumor-specific survival (the latter also being related to p53). For PTEN, its loss showed a higher association with biochemical recurrence and a worse prognosis, as well as Gleason score and tumor stage. Finally, ERG showed a strong association with biochemical recurrence.

If applied in specific situations, the use of these markers could guide the process of therapeutic decision making.

## Author contributions

AC, ÁB MW and BB drafted the manuscript and worked on the conception, design and interpretation of data. ÁB and LT selected articles, screened titles and abstracts, assessed study quality and extracted data. AC, ÁB, LT, PK, NM, CC, MW, IC, BB, GL, AS, MO and MT-M were involved in the interpretation and discussion of the results and critically revised the systematic review for important intellectual content. All authors approved the final version of the systematic review.

### Conflict of interest statement

The authors declare that the research was conducted in the absence of any commercial or financial relationships that could be construed as a potential conflict of interest.

## Abbreviations

PCa: Prostate cancer
PSA: Prostate-specific antigen
ISUP: International Society of Urological Pathology
IHC: Immunohistochemical
ERG: ETS-related gene
PTEN: Phosphatase and tensin homolog
TUR: Transurethral resection
MRI: Magnetic resonance imaging
EAU: European Association of Urology
AUA: American Urological Association.

## References

[1] Smith T Coburn M Sabiston Textbook of Surgery: The Biological Basis of Modern Surgical Practice. 20 th Edn. Elsevier (2017).

[2] Narayan VM Konety BR Warlick C. Novel biomarkers for prostate cancer: an evidence-based review for use in clinical practice. Int J Urol. (2017) 24:352–60. 10.1111/iju.13326 28345187

[3] Sharma S Zapatero-Rodriguez J O'Kennedy R. Prostate cancer diagnostics: clinical challenges and the ongoing need for disruptive and effective diagnostic tools. Biotechnol Adv. (2017) 35:135–49. 10.1016/j.biotechadv.2016.11.009 27939303

[4] Moyer VA Force USPST. Screening for prostate cancer: U.S. preventive services task force recommendation statement. Ann Intern Med. (2012) 157:120–34. 10.7326/0003-4819-157-2-201207170-00459 22801674

[5] Garisto JD Klotz L. Active surveillance for prostate cancer: how to do it right. Oncology (2017) 31:333–40, 45. 28512731

[6] Gleason DF Mellinger GT. Prediction of prognosis for prostatic adenocarcinoma by combined histological grading and clinical staging. J Urol. (1974) 111:58–64. 10.1016/S0022-5347(17)59889-4 4813554

[7] Gordetsky J Epstein J. Grading of prostatic adenocarcinoma: current state and prognostic implications. Diagn Pathol. (2016) 11:25. 10.1186/s13000-016-0478-2 26956509 PMC4784293

[8] Wilt TJ Brawer MK Jones KM Barry MJ Aronson WJ Fox S. Radical prostatectomy versus observation for localized prostate cancer. N Engl J Med. (2012) 367:203–13. 10.1056/NEJMoa1113162 22808955 PMC3429335

[9] Hamdy FC Donovan JL Lane JA Mason M Metcalfe C Holding P 10-Year outcomes after monitoring, surgery, or radiotherapy for localized prostate cancer. N Engl J Med. (2016) 375:1415–24. 10.1056/NEJMoa1606220 27626136

[10] Holmberg L Bill-Axelson A Steineck G Garmo H Palmgren J Johansson E. Results from the scandinavian prostate cancer group trial number 4: a randomized controlled trial of radical prostatectomy versus watchful waiting. J Natl Cancer Inst Monogr. (2012) 2012:230–3. 10.1093/jncimonographs/lgs025 23271778 PMC3540876

[11] Van der Kwast TH. Prognostic prostate tissue biomarkers of potential clinical use. Virchows Archiv (2014) 464:293–300. 10.1007/s00428-014-1540-7 24487790

[12] Jakobsen NA Hamdy FC Bryant RJ. Novel biomarkers for the detection of prostate cancer. J Clin. Urol. (2016) 9(Suppl. 2):3–10. 10.1177/2051415816656121 28344810 PMC5356177

[13] McGrath S Christidis D Perera M Hong SK Manning T Vela I. Prostate cancer biomarkers: are we hitting the mark? Prostate Int. (2016) 4:130–5. 10.1016/j.prnil.2016.07.002 27995111 PMC5153438

[14] Sharma P Zargar-Shoshtari K Pow-Sang JM. Biomarkers for prostate cancer: present challenges and future opportunities. Future Sci OA (2016) 2:FSO72. 10.4155/fso.15.72 28031932 PMC5137959

[15] Sulik M Expression of Ki-67 as a proliferation marker in prostate cancer. Polish Ann Med. (2011) 18:8 10.1016/S1230-8013(11)70019-4

[16] Missaoui N Abdelkarim SB Mokni M Hmissa S. Prognostic factors of prostate cancer in Tunisian men: immunohistochemical study. Asian Pac J Cancer Prev. (2016) 17:2655. 27268646

[17] Kim SH Park WS Park BR Joo J Joung JY Seo HK. PSCA, Cox-2, and Ki-67 are independent, predictive markers of biochemical recurrence in clinically localized prostate cancer: a retrospective study. Asian J Androl. (2017) 19:458–62. 10.4103/1008-682X.180798 27232854 PMC5507093

[18] Kudahetti S Fisher G Ambroisine L Foster C Reuter V Eastham J. p53 immunochemistry is an independent prognostic marker for outcome in conservatively treated prostate cancer. BJU Int. (2009) 104:20–4. 10.1111/j.1464-410X.2009.08407.x 19239456

[19] Griend DV Molecular Biology of Prostate Cancer. Waltham, MA: UpToDate (2017).

[20] Klumb CE Júnior GBC Avaliação dos métodos de detecção das alterações do gene e proteína p53 nas neoplasia linfóides. Rev Bras Hematol Hemoter. (2002) 24:111–25. 10.1590/S1516-84842002000200008

[21] Kumari R Sen N Das S Tumour suppressor p53: understanding the molecular mechanisms inherent to cancer. Curr. Sci. (2014) 107:786–94.

[22] Adamo P Ladomery M. The oncogen ERG: a key factor in prostate cancer. Oncogene (2015) 35:403–14. 10.1038/onc.2015.109 25915839

[23] Ferraldeschi R Nava Rodrigues D Riisnaes R Miranda S Figueiredo I Rescigno P Ravi P. PTEN protein loss and clinical outcome from castration-resistant prostate cancer treated with abiraterone acetate. Eur Urol. (2015) 67:795–802. 10.1016/j.eururo.2014.10.027 25454616 PMC4410287

[24] Gurel B Iwata T Koh CM Jenkins RB Lan F Van Dang C. Nuclear MYC protein overexpression is an early alteration in human prostate carcinogenesis. Mod Pathol. (2008) 21:1156–67. 10.1038/modpathol.2008.111 18567993 PMC3170853

[25] Udager AM DeMarzo AM Shi Y Hicks JL Cao X Siddiqui J. Concurrent nuclear ERG and MYC protein overexpression defines a subset of locally advanced prostate cancer: potential opportunities for synergistic targeted therapeutics. Prostate (2016) 76:845–53. 10.1002/pros.23175 27159573 PMC4975940

[26] Moher D Liberati A Tetzlaff J Altman D Group TP Preferred reporting items for systematic reviews and meta-analyses: the PRISMA statement. PLoS Med. (2009) 6:e1000097 10.1371/journal.pmed.1000097 19621072 PMC2707599

[27] Lotan TL Gurel B Sutcliffe S Esopi D Liu W Xu J. PTEN protein loss by immunostaining: analytic validation and prognostic indicator for a high risk surgical cohort of prostate cancer patients. Clin Cancer Res. (2011) 17:6563–73. 10.1158/1078-0432.CCR-11-1244 21878536 PMC3195839

[28] Murphy SJ Karnes RJ Kosari F Castellar BE Kipp BR Johnson SH. Integrated analysis of the genomic instability of PTEN in clinically insignificant and significant prostate cancer. Modern Pathol. (2016) 29:143–56. 10.1038/modpathol.2015.136 26612463

[29] Shah RB Bentley J Jeffery Z DeMarzo AM. Heterogeneity of PTEN and ERG expression in prostate cancer on core needle biopsies: implications for cancer risk stratification and biomarker sampling. Hum Pathol. (2015) 46:698–706. 10.1016/j.humpath.2015.01.008 25724568

[30] Nagao K Yamamoto Y Hara T Komatsu H Inoue R Matsuda K. Ki67 and BUBR1 may discriminate clinically insignificant prostate cancer in the PSA range <4 ng/ml. Jpn J Clin Oncol. (2011) 41:555–64. 10.1093/jjco/hyq233 21233104

[31] Bubendorf L Tapia C Gasser TC Casella R Grunder B Moch H. Ki67 labeling index in core needle biopsies independently predicts tumor-specific survival in prostate cancer. Hum Pathol. (1998) 29:949–54. 10.1016/S0046-8177(98)90199-X 9744310

[32] Zellweger T Gunther S Zlobec I Savic S Sauter G Moch H Tumour growth fraction measured by immunohistochemical staining of Ki67 is an independent prognostic factor in preoperative prostate biopsies with small-volume or low-grade prostate cancer. Int J Cancer (2009) 124:2116–23. 10.1002/ijc.24174 19117060

[33] Aldaoud N Abdo N Al Bashir S Alqudah M Marji N Alzou'bi H. Prostate cancer in Jordanian-Arab population: ERG status and relationship with clinicopathologic characteristics. Virchows Arch. (2017). 471:753–9. 10.1007/s00428-017-2160-9 28550496

[34] Jiang FN He HC Zhang YQ Yang DL Huang JH Zhu YX. An integrative proteomics and interaction network-based classifier for prostate cancer diagnosis. PLoS ONE (2013) 8:e63941. 10.1371/journal.pone.0063941 23737958 PMC3667836

[35] Guedes LB Tosoian JJ Hicks J Ross AE Lotan TL. PTEN loss in gleason score 3 + 4 = 7 prostate biopsies is associated with nonorgan confined disease at radical prostatectomy. J Urol. (2017) 197:1054–9. 10.1016/j.juro.2016.09.084 27693448

[36] Zeng W Sun H Meng F Liu Z Xiong J Zhou S. Nuclear C-MYC expression level is associated with disease progression and potentially predictive of two year overall survival in prostate cancer. Int J Clin Exp Pathol. (2015) 8:1878–88. 25973080 PMC4396295

[37] Lotan TL Carvalho FL Peskoe SB Hicks JL Good J Fedor H. PTEN loss is associated with upgrading of prostate cancer from biopsy to radical prostatectomy. Mod Pathol. (2015) 28:128–37. 10.1038/modpathol.2014.85 24993522 PMC4282985

[38] Leinonen KA Saramaki OR Furusato B Kimura T Takahashi H Egawa S. Loss of PTEN is associated with aggressive behavior in ERG-positive prostate cancer. Cancer Epidemiol Biomarkers Prev. (2013) 22:2333–44. 10.1158/1055-9965.EPI-13-0333-T 24083995 PMC4086660

[39] Fontugne J Lee D Cantaloni C Barbieri CE Caffo O Hanspeter E. Recurrent prostate cancer genomic alterations predict response to brachytherapy treatment. Cancer Epidemiol Biomarkers Prevent. (2014) 23:594–600. 10.1158/1055-9965.EPI-13-1180 24515272 PMC4083705

[40] Kim SH Joung JY Lee GK Hong EK Kang KM Yu A. Overexpression of ERG and wild-type PTEN are associated with favorable clinical prognosis and low biochemical recurrence in prostate cancer. PLoS ONE (2015) 10:e0122498. 10.1371/journal.pone.0122498 25897494 PMC4405492

[41] Suh JH Park JW Lee C Moon KC. ERG immunohistochemistry and clinicopathologic characteristics in Korean prostate adenocarcinoma patients. Korean J Pathol. (2012) 46:423–8. 10.4132/KoreanJPathol.2012.46.5.423 23136568 PMC3490118

[42] Kron K Liu L Trudel D Pethe V Trachtenberg J Fleshner N. Correlation of ERG expression and DNA methylation biomarkers with adverse clinicopathologic features of prostate cancer. Clin Cancer Res. (2012) 18:2896–904. 10.1158/1078-0432.CCR-11-2901 22452941

[43] Szász AM Majoros A Rosen P Srivastava S Dobi A Szendroi A. Prognostic potential of ERG (ETS-related gene) expression in prostatic adenocarcinoma. Int Urol Nephrol. (2013) 45:727–33. 10.1007/s11255-013-0406-2 23686669 PMC3691333

[44] Schaefer G Mosquera JM Ramoner R Park K Romanel A Steiner E. Distinct ERG rearrangement prevalence in prostate cancer: higher frequency in young age and in low PSA prostate cancer. Prostate Cancer Prostatic Dis. (2013) 16:132–8. 10.1038/pcan.2013.4 23381693 PMC3655380

[45] Brooks JD Wei W Hawley S Auman H Newcomb L Boyer H. Evaluation of ERG and SPINK1 by immunohistochemical staining and clinicopathological outcomes in a multi-institutional radical prostatectomy cohort of 1067 patients. PLoS ONE (2015) 10:e0132343. 10.1371/journal.pone.0132343 26172920 PMC4501723

[46] Silva MP Barros-Silva JD Ersvær E Kildal W Hveem TS Pradhan M. Cancer prognosis defined by the combined analysis of 8q, PTEN and ERG. Transl Oncol. (2016) 9:575–82. 10.1016/j.tranon.2016.08.005 27916292 PMC5143339

[47] Lotan TL Wei W Morais CL Hawley ST Fazli L Hurtado-Coll A. PTEN loss as determined by clinical-grade immunohistochemistry assay is associated with worse recurrence-free survival in prostate cancer. Eur Urol Focus (2016) 2:180–8. 10.1016/j.euf.2015.07.005 27617307 PMC5014432

[48] Lahdensuo K Erickson A Saarinen I Seikkula H Lundin J Lundin M. Loss of PTEN expression in ERG-negative prostate cancer predicts secondary therapies and leads to shorter disease-specific survival time after radical prostatectomy. Modern Pathol. (2016) 29:1565–74. 10.1038/modpathol.2016.154 27562498

[49] Ahearn TU Pettersson A Ebot EM Gerke T Graff RE Morais CL. A prospective investigation of PTEN loss and ERG expression in lethal prostatecancer. J Natl Cancer Inst. (2016) 108:1–9. 10.1093/jnci/djv346 26615022 PMC4862436

[50] Prowatke I Devens F Benner A Gröne EF Mertens D Gröne HJ. Expression analysis of imbalanced genes in prostate carcinoma using tissue microarrays. Br J Cancer (2007) 96:82–8. 10.1038/sj.bjc.6603490 17146477 PMC2360197

[51] Chevrier M Bobbala D Villalobos-Hernandez A Khan MG Ramanathan S Saucier C. Expression of SOCS1 and the downstream targets of its putative tumor suppressor functions in prostate cancer. BMC Cancer (2017) 17:157. 10.1186/s12885-017-3141-8 28235401 PMC5326496

[52] Zellweger T Ninck C Bloch M Mirlacher M Koivisto PA Helin HJ. Expression patterns of potential therapeutic targets in prostate cancer. Int J Cancer (2005) 113:619–28. 10.1002/ijc.20615 15472903

[53] Zellweger T Ninck C Mirlacher M Annefeld M Glass AG Gasser TC. Tissue microarray analysis reveals prognostic significance of syndecan-1 expression in prostate cancer. Prostate (2003) 55:20–9. 10.1002/pros.10209 12640657

[54] Bubendorf L Sauter G Moch H Jordan P Blochlinger A Gasser TC. Prognostic significance of Bcl-2 in clinically localized prostate cancer. Am J Pathol. (1996) 148:1557–65. 8623924 PMC1861580

[55] Xu B Chevarie-Davis M Chevalier S Scarlata E Zeizafoun N Dragomir A. The prognostic role of ERG immunopositivity in prostatic acinar adenocarcinoma: a study including 454 cases and review of the literature. Hum Pathol. (2014) 45:488–97. 10.1016/j.humpath.2013.10.012 24406017

[56] Hoogland AM Jenster G van Weerden WM Trapman J van der Kwast T Roobol MJ ERG immunohistochemistry is not predictive for PSA recurrence, local recurrence or overall survival after radical prostatectomy for prostate cancer. Mod Pathol. (2012) 25:471–9. 10.1038/modpathol.2011.176 22080055

[57] Hagen RM Adamo P Karamat S Oxley J Aning JJ Gillatt D. Quantitative analysis of ERG expression and its splice isoforms in formalin-fixed, paraffin-embedded prostate cancer samples: association with seminal vesicle invasion and biochemical recurrence. Am J Clin Pathol. (2014) 142:533–40. 10.1309/AJCPH88QHXARISUP 25239421

[58] Weinmann S Van Den Eeden SK Haque R Chen C Richert-Boe K Schwartzman J. Immunohistochemical expression of ERG in the molecular epidemiology of fatal prostate cancer study. Prostate (2013) 73:1371–7. 10.1002/pros.22684 23661613 PMC3745520

[59] Graff RE Meisner A Ahearn TU Fiorentino M Loda M Giovannucci EL. Pre-diagnostic circulating sex hormone levels and risk of prostate cancer by ERG tumour protein expression. Br J Cancer (2016) 114:939–44. 10.1038/bjc.2016.61 26986253 PMC4984801

[60] Halvorsen OJ Haukaas SA Akslen LA. Combined loss of PTEN and p27 expression is associated with tumor cell proliferation by Ki-67 and increased risk of recurrent disease in localized prostate cancer. Clin Cancer Res. (2003) 9:1474–9. 12684422

[61] Font-Tello A Juanpere N de Muga S Lorenzo M Lorente JA Fumado L. Association of ERG and TMPRSS2-ERG with grade, stage, and prognosis of prostate cancer is dependent on their expression levels. Prostate (2015) 75:1216–26. 10.1002/pros.23004 25939480

[62] Taris M Irani J Blanchet P Multigner L Cathelineau X Fromont G. ERG expression in prostate cancer: the prognostic paradox. Prostate (2014) 74:1481–7. 10.1002/pros.22863 25175352

[63] Nishijima J Hara T Ikemoto K Oga A Kobayashi K Kawai Y. Clinical significance of ERG rearrangement subtype and its association with increased p53 expression in Japanese and German prostate cancer. Neoplasma (2015) 62:278–87. 10.4149/neo_2015_033 25591593

[64] Tosoian JJ Almutairi F Morais CL Glavaris S Hicks J Sundi D. Prevalence and prognostic significance of PTEN Loss in african-american and european-american men undergoing radical prostatectomy. Eur Urol. (2017) 71:697–700. 10.1016/j.eururo.2016.07.026 27477529 PMC5274596

[65] Inoue T Segawa T Shiraishi T Yoshida T Toda Y Yamada T. Androgen receptor, Ki67, and p53 expression in radical prostatectomy specimens predict treatment failure in Japanese population. Urology (2005) 66:332–7. 10.1016/j.urology.2005.02.028 16098362

[66] Osman I Drobnjak M Fazzari M Ferrara J Scher HI Cordon-Cardo C. Inactivation of the p53 pathway in prostate cancer: impact on tumor progression. Clin Cancer Res. (1999) 5:2082–8. 10473090

[67] Epstein JI Chan DW Sokoll LJ Walsh PC Cox JL Rittenhouse H. Nonpalpable stage T1c prostate cancer: prediction of insignificant disease using free/total prostate specific antigen levels and needle biopsy findings. J Urol. (1998) 160(6 Pt 2):2407–11. 10.1097/00005392-199812020-00008 9817393

[68] Bokhorst LP Valdagni R Rannikko A Kakehi Y Pickles T Bangma CH. A decade of active surveillance in the PRIAS study: an update and evaluation of the criteria used to recommend a switch to active treatment. Eur Urol. (2016) 70:954–60. 10.1016/j.eururo.2016.06.007 27329565

[69] Cuzick J Yang ZH Fisher G Tikishvili E Stone S Lanchbury JS. Prognostic value of PTEN loss in men with conservatively managed localised prostate cancer. Br J Cancer (2013) 108:2582–9. 10.1038/bjc.2013.248 23695019 PMC3694239

[70] Schröder FH Hugosson J Roobol MJ Tammela TL Ciatto S Nelen V. Screening and prostate-cancer mortality in a randomized European study. N Engl J Med. (2009) 360:1320–8. 10.1056/NEJMoa0810084 19297566

[71] Andriole GL Crawford ED Grubb RL Buys SS Chia D Church TR. Mortality results from a randomized prostate-cancer screening trial. N Engl J Med. (2009) 360:1310–9. 10.1056/NEJMoa0810696 19297565 PMC2944770

[72] Martin RM Donovan JL Turner EL Metcalfe C Young GJ Walsh EI. Effect of a low-intensity PSA-based screening intervention on prostate cancer mortality: the CAP randomized clinical trial. JAMA (2018) 319:883–95. 10.1001/jama.2018.0154 29509864 PMC5885905

[73] Chen RC Basak R Meyer AM Kuo TM Carpenter WR Agans RP. Association between choice of radical prostatectomy, external beam radiotherapy, brachytherapy, or active surveillance and patient-reported quality of life among men with localized prostate cancer. JAMA (2017) 317:1141–50. 10.1001/jama.2017.1652 28324092 PMC6284802

[74] Sanda MG Cadeddu JA Kirkby E Chen RC Crispino T Fontanarosa J Clinically localized prostate cancer: AUA/ASTRO/SUO guideline. Part I: risk stratification, shared decision making, and care options. J Urol. (2018) 199:683–90. 10.1016/j.juro.2017.11.095 29203269

[75] Maurice MJ Kim SP Abouassaly R. Current status of prostate cancer diagnosis and management in the United States. JAMA Oncol. (2016) 2:1505–7. 10.1001/jamaoncol.2016.1785 27356204

